# Transcriptomic Analysis Reveals Genetic Cross-Talk between Periodontitis and Hypothyroidism

**DOI:** 10.1155/2022/5736394

**Published:** 2022-04-11

**Authors:** Bin Yan, Fukai Ren, Wei Shang, Xiaoyan Gong

**Affiliations:** ^1^Department of Stomatology, Changzhi Medical College, Changzhi, 046000 Shanxi Province, China; ^2^Department of Stomatology, Heping Hospital Affiliated to Changzhi Medical College, Changzhi, 046000 Shanxi Province, China

## Abstract

**Background:**

Aim of this bioinformatics study based on transcriptomic analysis was to reveal the cross-talk between periodontitis (PD) and hypothyroidism (HT).

**Methods:**

The gene expression datasets GSE18152 and GSE176153 of HT and GSE10334, GSE16134, and GSE173078 of PD were downloaded through the Gene Expression Omnibus (GEO) database. Differential Expression Genes (DEG) between cases and controls in each microarray were assessed by using the “limma” (linear models for microarray data) R package (|log2 fold change (FC)| >0 and *P*-value <0.05). To analyze the cross-talk effect between HT and PD, the intersection of DEG of HT and PD was selected. To investigate the biological function of cross-talk genes, the gene ontology (GO) functional enrichment analysis and Kyoto Encyclopedia of Genes and Genomes (KEGG) pathway analysis were applied. Protein-Protein Interaction (PPI) network was constructed using Cytoscape software. Top 10 cross-talk genes were screened, and the expression values of these 10 genes were extracted. ROC analysis was performed by using the pROC package and GGplot2 package of R language to predict the classification accuracy.

**Results:**

The overlapping DEG between HT and PD were 107 cross-talk genes. The results revealed that developmental process (*P*-value =1.06E-21) was the most significantly enriched biological process, followed by cell differentiation (*P*-value =8.49E-18) and immune system process (*P*-value =6.78E-11). KEGG analysis showed that Complement and coagulation cascades (*P*-value =2.29E-05), Hematopoietic cell lineage (*P*-value =2.66E-05), Phospholipase D signaling pathway (*P*-value =0.034367878) and Chemokine signaling pathway (*P*-value =0.04946333) were significantly enriched. The top 10 genes with most connections were LCE1B, LCE2B, LCE2A, LCE2C, LCE1C, LCE1F, ITGAM, C1QB, TREM2, and CD19. The AUC values of the two datasets of HT were both greater than 65% (GSE18152 = 81.42%, GSE176153 = 68.75%). AUC values of three datasets of PD were all greater than 60% (GSE10334 = 69.23%, GSE16134 = 73.72%, GSE173078 = 81.6%).

**Conclusions:**

A genetic cross-talk between HT and PD was detected, whereby LCE family genes appeared to play the most important role.

## 1. Background

Thyroid dysfunction is highly prevalent, as it is one of the leading endocrine disorders; therefore, thyroid diseases are a global health problem [1]. Thereby, hypothyroidism (HT) is the most common thyroid dysfunction, with a prevalence range between 0 and 7% across European and US populations [2]. Depending on its cause, primary, secondary, tertiary, and peripheral HT can be distinguished. The symptoms of HT can be multifarious; most common clinical signs are fatigue, lethargy, cold intolerance, weight gain, constipation, change in voice, and dry skin, what can potentially vary between different age and gender groups [2].

Periodontal diseases (PD), i.e., the inflammatory, biofilm-related destruction of the tooth surrounding tissues, represent a multifactorial disease [3]. Thus, periodontal-systemic interaction is an issue of scientific interest during the past decades. Since the 70s of the last century, changes in periodontal tissues related to HT were observed [4]. It has been reported that a reduction in serum levels of thyroid hormones, as clinically present in HT, increases the bone loss related to periodontal inflammation in animal model [5]. Meanwhile, a clinical relationship between PD and HT appears reasonable and is supported by the literature, although the body of evidence is still weak [6]. This is supported by several findings in recent literature; on the one hand, periodontal therapy was found to positively influence the thyroid status, whereby Interleukin 6 and TNF alpha were revealed as potential key players [7]. Moreover, Shcherba et al. showed that HT would be related to PD development and increased destruction of connective tissue [8]. These data lead to the conclusion of a potential interlink between PD and HT, although the molecular mechanisms and pathophysiological interplay are not fully understood, yet.

Recently, bioinformatics analysis was able to reveal different cross-talk mechanisms on transcriptomic level for periodontal-systemic interactions, e.g., between PD and Alzheimer's disease [9], PD and atherosclerosis [10], PD and oral cancer [11], or periimplantitis and rheumatoid arthritis [12]. This reasonable methodical approach has the potential to get a deeper understanding of the topic and to generate further hypotheses for clinical research questions. Therefore, this current study applied bioinformatics analysis based on publicly available datasets with the aim to reveal the cross-talk between PD and HT. During the analysis process, differential expressed genes between the two diseases should be detected and analyzed regarding their predictive potential as cross-talk genes between PD and HT. It was hypothesized that PD and HT share several cross-talk genes and related pathways on transcriptomic level. Thereby, inflammation-related genes might be of highest importance in this context.

## 2. Material and Methods

### 2.1. Data Search and Extraction

The gene expression datasets of hypothyroidism (HT) and periodontal diseases (PD) were downloaded through the Gene Expression Omnibus (GEO) database (http://www.ncbi.nlm.nih.gov/). We systematically searched the microarray studies by using the terms: “hypothyroidism,” periodontitis,” and “Homo sapiens.”

For HT, samples with whole blood were available for analysis. Hypothyroidism (HT) patients or Congenital Hypothyroidism (CH) patients acted as cases, and the control (CTL) groups or healthy patients acted as controls for analysis. Therefore, GSE18152 and GSE176153 of HT were screened and included into analysis (Table 1).

For PD, samples with gingival tissue were available for analysis. Both chronic and aggressive PD patients acted as cases, while control groups or healthy patients acted as controls. Finally, GSE10334, GSE16134, and GSE173078 of PD were screened and included (Table 1). There were two microarray datasets and one high throughput sequencing dataset for PD. One microarray dataset and one high throughout sequencing dataset for HT were included.

### 2.2. Dataset Preparation

First, the high throughput sequencing dataset, the gene expression matrix, and related annotation platform for each dataset were downloaded from GEO database. Corresponding platforms were used to map the microarray probes to gene symbols. If multiple probes mapped to the same symbol, the mean value was adopted. There was no gene information for the platform GPL5114, which was the corresponding annotation document of GSE18152. Based on the hg19 genome of the UCSC table tools (http://genome.ucsc.edu/), the samples of GPL5114 were mapped to genes. With the transformed platform of GPL5114, the samples of GSE18152 were mapped to genes. Second, when the number of zero in the cases or controls for a gene exceeded half of total samples, the gene was deleted from the expression matrix.

### 2.3. Differential Expression Analysis

For the microarray datasets in PD and HT, the Differential Expression Genes (DEG) were determined between cases and controls in each microarray by using the “limma” (linear models for microarray data) R package. For the high throughput sequencing datasets in PD and HT, the DEG were determined between cases and controls in each dataset by using the “DEseq2” R package. The |log2 fold change (FC)| >0 and *P*-value <0.05 were regarded as the cut-off criteria to determine DEG.

### 2.4. Cross-Talk Genes

To identify the potential cross-talk genes between HT and PD, DEG of each dataset for HT were combined, and the combined DEG acted as the final DEG for HT. Meanwhile, DEG of each dataset for PD were combined, and the combined DEG acted as the final DEG for PD. To analyze the cross-talk effect between HT and PD, the intersection of DEG of HT and PD was selected, and these intersection genes were considered the potential cross-talk genes of HT and PD.

To investigate the biological function of cross-talk genes, the gene ontology (GO) functional enrichment analysis and Kyoto Encyclopedia of Genes and Genomes (KEGG) pathway analysis were applied. We uploaded the cross-talk genes to investigate the potential functions with gProfiler (https://biit.cs.ut.ee/gprofiler/gost). *P*-value <0.05 and false discovery rate (FDR) <0.05 were regarded as the cut-off criteria.

### 2.5. Protein-Protein Interaction (PPI) Network Analysis for Cross-Talk Genes

To analyze the role of cross-talk genes in biological networks, the protein-protein interactions were applied. The cross-talk genes were uploaded to the STRING database (http://www.string-db.org/), then the PPI network of cross-talk genes was constructed using Cytoscape software. In the PPI network, each node represents a gene or protein, and the edge between nodes represents the interaction of the molecules. Hub genes are usually deemed to be functionally critical and highly interconnected with other genes. Cytohubba plugin of Cytoscape was applied to explore the hub genes. Cytohubba identified important nodes/hubs and fragile motifs in an interactome network by several topological algorithms including Degree, Edge Percolated Component (EPC), Maximum Neighborhood Component (MNC), Density of Maximum Neighborhood Component (DMNC), Maximal Clique Centrality (MCC), and centralities based on shortest paths, such as Bottleneck (BN), EcCentricity, Closeness, Radiality, Betweenness, and Stress.

### 2.6. Hub Genes Validation Study

In the analysis of network topological properties of cross-talk genes, the larger the MCC of the gene in the network is, the more important the gene acts in the network. Top 10 cross-talk genes were screened by the MCC, and the expression values of these 10 genes in each dataset of HT and PD were extracted. Based on the gene expression value of genes in cases and controls, ROC analysis was performed by using the pROC package and GGplot2 package of R language to predict the classification accuracy.

## 3. Results

### 3.1. Identification of DEG

**Figure 1:**
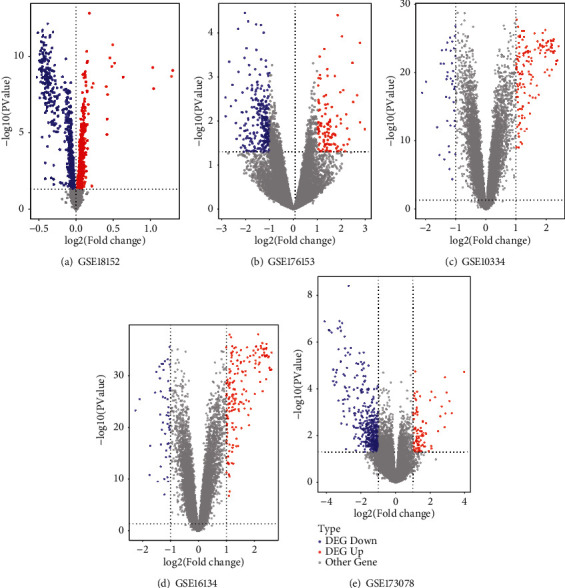
Volcano plots of HT ((a) and (b)) and PD ((c)–(e)). The abscissa is log2FoldChange and the ordinate is -log10 (*P*-value). The blue dots represent down-regulated genes, the red dots represent up-regulated genes, and the gray dots represent genes that are not differentially expressed.

All datasets were analyzed separately in the process of differential expression analysis. The DEG were screened out according to the cut-off criteria. For the GSE10334, GSE16134, GSE173078, and GSE17615, the genes with *p*-value <0.05 and |log2(FC)| >1 were the DEG, while Log2(FC) >1 were the up-regulated genes and log2(FC) < -1 were the down-regulated genes. For GSE18152, the genes with *p*-value <0.05 and |log2(FC)| >0 were the DEG, whereby Log2(FC) >0 were up-regulated and log2(FC) < -9 were down-regulated. The DEG counts are listed in Table 2. The volcano plots of the datasets are shown in Figure 1.

### 3.2. Cross-Talk Genes between HT and PD

**Figure 2:**
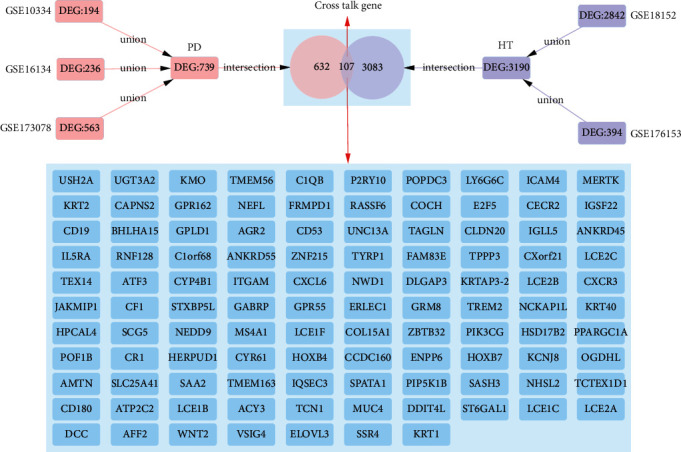
Cross-talk genes between HT and PD.

**Figure 3:**
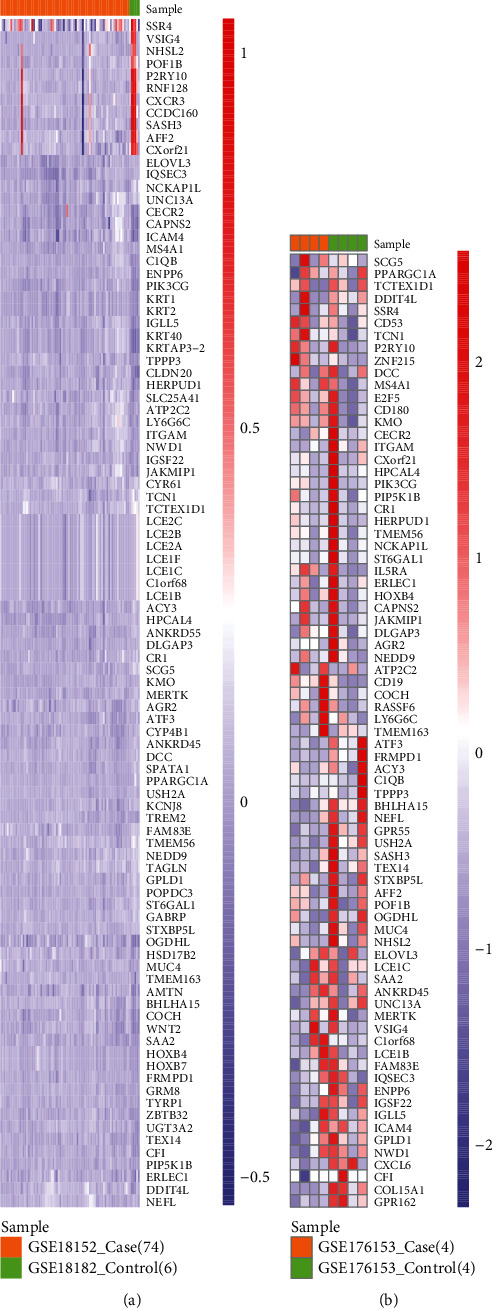
Expression level of cross-talk gene in HT.

**Figure 4:**
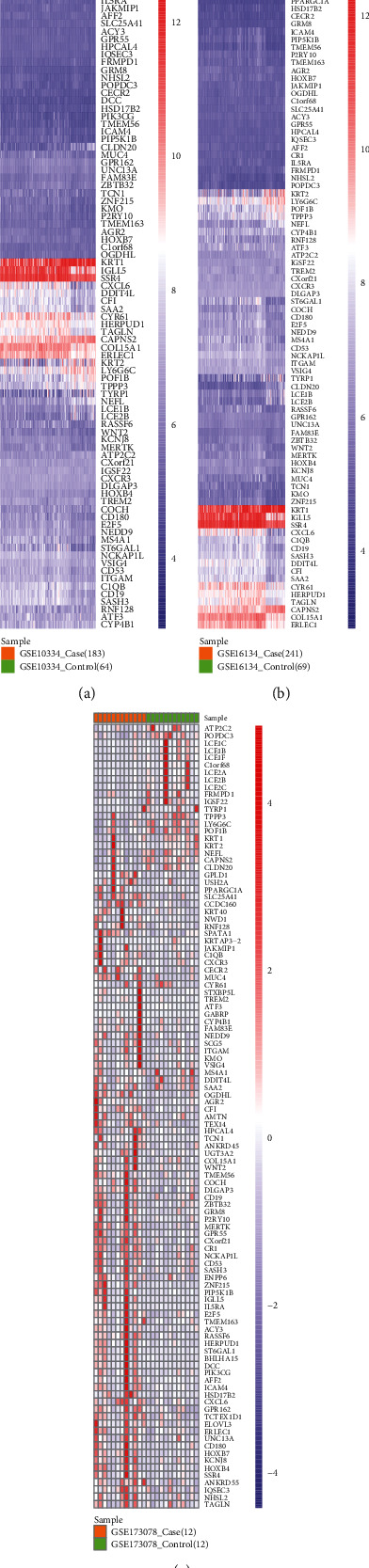
Expression level of cross-talk gene in PD.

After differential expressed analysis, 3190 DEG were obtained for HT and 739 DEG for PD. The overlapping DEG between HT and PD were the cross-talk genes, whereby 107 cross-talk genes were acquired (Figure 2). To observe the changing trend of the expression level of cross-talk genes in HT and PD, we used pheatmap package of R language to display the expression of cross-talk genes in HT and PD (Figures 3 and 4 ).

### 3.3. Biological Function Analysis of Cross-Talk Genes

**Figure 5:**
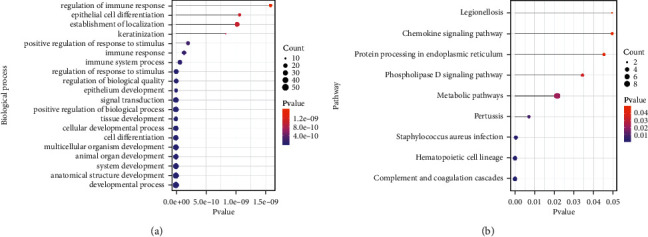
The GO biological process (a) and (b) pathway enriched by cross-talk genes. The role of cross-talk gene in biological networks.

We uploaded the 107 cross-talk genes to perform the GO (including biological process, molecular function, and cellular component) analysis and KEGG analysis. The results revealed that developmental process (GO:0032502; *P*-value =1.06E-21) was the most significantly enriched biological process, followed by cell differentiation (GO:0030154; *P*-value =8.49E-18) and immune system process (GO:0002376; *P*-value =6.78E-11) (Figure 5(a)). Furthermore, KEGG pathway enrichment analysis showed that Complement and coagulation cascades (KEGG:04610; *P*-value =2.29E-05), Hematopoietic cell lineage (KEGG:04640; *P*-value =2.66E-05), Phospholipase D signaling pathway (KEGG:04072; *P*-value =0.034367878), and Chemokine signaling pathway (KEGG:04062; *P*-value =0.04946333) were significantly enriched (Figure 5(b)).

**Figure 6:**
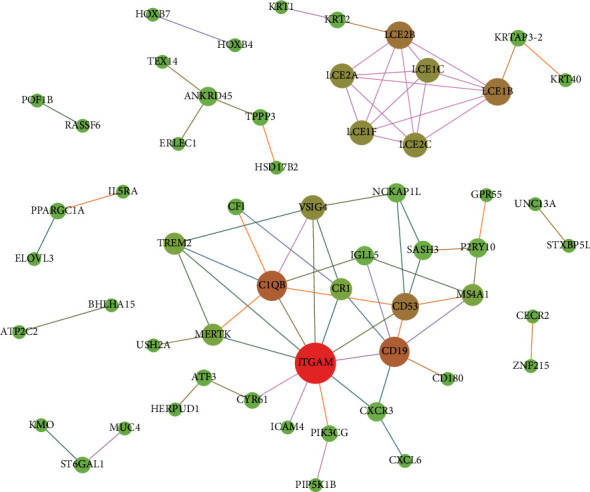
The PPI network of cross-talk genes. The more other nodes a node is connected to, the larger the node will be. The thicker the edge between the two nodes, the larger their combined score will be.

STRING database was used to perform PPI network analysis of the cross-talk genes and 73 PPI for cross-talk genes were acquired. Cytoscape software was adopted to visualize the PPI network (Figure 6). In the PPI analysis, the hub genes may play pivotal physiological regulatory roles. Cytohubba was applied to identify the hub genes, and the top 10 genes with most connections were identified (LCE1B, LCE2B, LCE2A, LCE2C, LCE1C, LCE1F, ITGAM, C1QB, TREM2, and CD19).  Table 3 shows the topological characteristics of top 10 genes in PPI network.

### 3.4. ROC Analysis for Hub Genes

**Figure 7:**
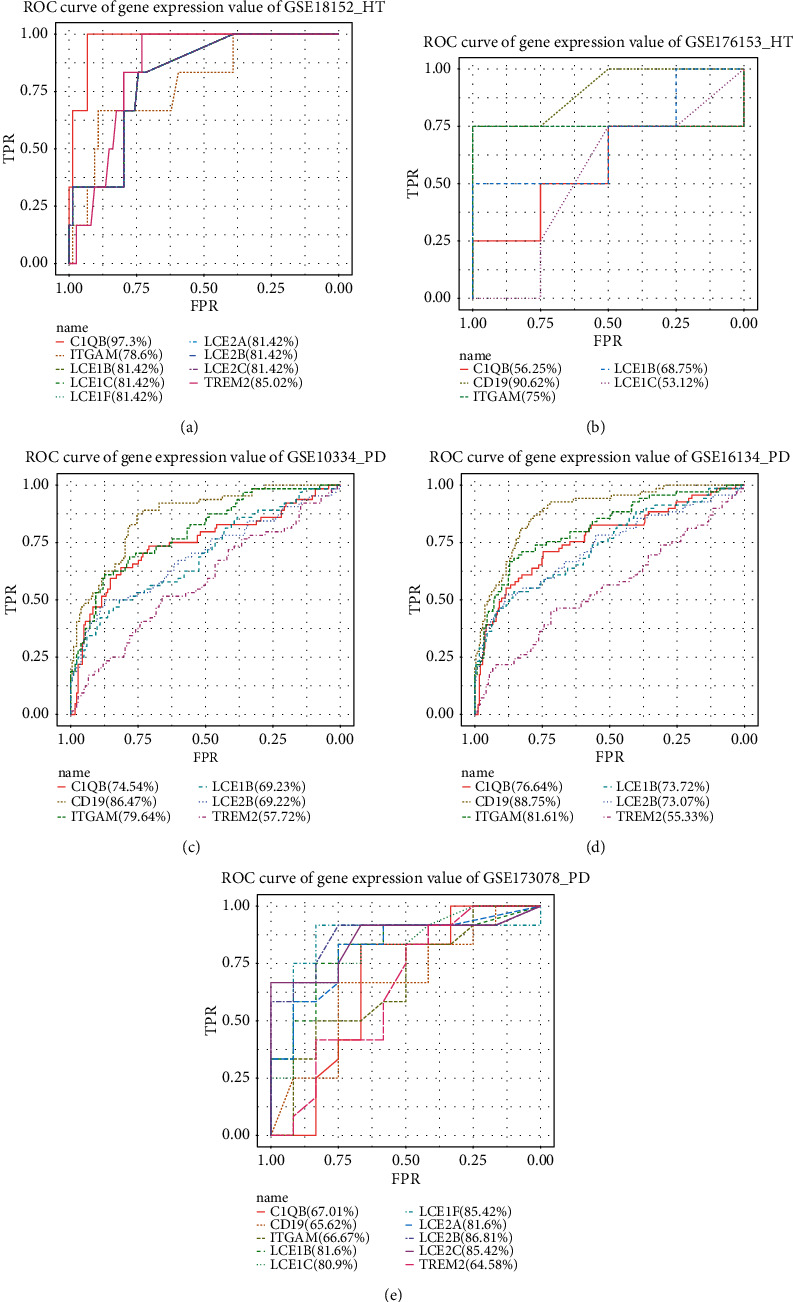
Prediction of hub gene for HT ((a) and (b)) and PD ((c)–(e)).

In order to analyze the prediction effect of hub genes on diseases, the sample values of 10 hub genes in each dataset were extracted. Based on the cases and controls, ROC analysis was performed on the obtained datasets by using the pROC package and GGplot2 package of R language (Figure 7).

The results showed that LCE1B appeared in both HT and PD datasets. The AUC values of the two datasets of HT were both greater than 65% (GSE18152 = 81.42%, GSE176153 = 68.75%). AUC values of three datasets of PD were all greater than 60% (GSE10334 = 69.23%, GSE16134 = 73.72%, GSE173078 = 81.6%). Although the prediction effect of the 10 hub genes was inconsistent across all datasets in HT and PD, the genes interact with each other to jointly influence disease progression. LCE1B interacts with other hub genes (LCE2B, LCE2A, LCE2C, LCE1C, and LCE1F), and the AUC of other hub genes (LCE2B, LCE2A, LCE2C, LCE1C, and LCE1F) in HT and PD is greater than 65%. The results showed that LCE family genes play a cross-talk role in PD and hypothyroidism.

## 4. Discussion

Although the body of evidence is low, HT and PD appear to be related to each other [6]. Thereby, one potential issue of importance is the bone metabolism; thyroid hormones are crucial for bone maintenance, whereby respective dysfunctions like HT affect bone structure [13]. Because PD walks along with progressive bone loss, this relationship appears reasonable [5]. In this context, it is not surprising that patients with HT show a pronounced destruction of respective connective tissues [8]. The second approach is the influence of HT on the PD-related immune response; in experimental PD, thyroid dysfunction was found to foster cytokine imbalance and severity of inflammation [14]. In addition to that, a potential role of vitamin D in the interplay between PD and HT was reported [15]. While those mechanisms appear conceivable, the underlying mechanisms remain poorly understood. This current study found several potential cross-talk genes and related pathways between PD and HT, which might serve as a basis for future research in the field.

Based on the ROC analysis, the LCE family was found to be the most relevant cross-talk genes in the interplay between PD and HT. Late cornified envelope (LCE) protein is important for epidermal differentiation, especially with regard to keratinocytes [16]. The LCE cluster includes different conserved genes, which encode stratum corneum proteins [17]. Until now, the LCE family was neither found to be associated with PD nor with HT. Accordingly, specific hypotheses on the relevance of LCE genes in the interplay between those two diseases cannot be formed and remain speculative. LCE is reported to be related to psoriasis and psoriatic arthritis [18]. Thereby, the LCE, although primarily related to group 3, which was not identified in the current study, is involved in inflammation repair of the skin [19]. Thus, LCE was related to (auto-) inflammation in context of psoriasis [20]. Psoriasis is associated with PD, although evidence is somewhat weak [21]. As a comorbidity of psoriasis, PD has an immunomodulatory effect on psoriatic exacerbation [22]. Moreover, thyroid autoimmunity was also found to be related to psoriasis [23]. Considering this, the potential role of LCE family as cross-talk genes between PD and HT would most likely indicate an increase of (auto-) immunity; this is supported by the upper mentioned fact that the thyroid dysfunction would foster cytokine imbalance and severity of periodontal inflammation [14]. Furthermore, the biological processes immune system process and complement, which were identified as potentially relevant in the current study, would be in line with this hypothesis.

Most of the further identified potential cross-talk genes support the hypotheses of increased (auto-) inflammation to be relevant in the interplay between PD and HT. ITGAM was found to be up-regulated in gingival tissues of periodontal diseased patients [24]. Similarly, the complement C1QB was related to periodontal inflammation [25, 26], while no results for HT could be found in this respect. CD19 was revealed to be strongly associated with pro-inflammatory cytokines in PD [27]. Another study showed that CD19 positive B cells were one major B cell component in PD [28]. Similarly, CD19 was related to autoimmunity of the thyroid [29]. All of these findings could support the (auto-) immune relationship between PD and HT. Finally, triggering receptor expressed on myeloid cells-2 (TREM2) was found to be a cross-talk gene in the current study. TREM2 is an important stimulator of osteoclast differentiation and bone loss in PD [30]. Furthermore, TREM2 is crucial in osteoclastogenesis within PD microenvironment [31]. TREM2 is regulated by thyroid hormones [32]. Thus, the relationship between PD and HT based on bone metabolism appears plausible.

This current bioinformatics study was comprehensive and addressed a topic of clinical relevance. The underlying approach of analyzing a systemic disease and its relationship to periodontal diseases is reasonable and was repeatedly applied in previous studies [9–12]. The main limitation is the absence of a clinical validation, making all of the discussions and derived conclusions speculative. Different heterogeneous patients and tissues were examined, what limits the generalizability of the findings. Similarly, a high variety of results was found, especially regarding potentially related pathways and processes. Based on the limited body of literature, the importance of those findings is difficult to estimate and a deeper discussion would be largely speculative at the moment. Although the methodology appears reasonable, the findings are only on transcriptomic level and can only form a basis for future research in the field; the hypotheses within this current study can be seen as a basis for clinical studies.

## 5. Conclusions

A genetic cross-talk between HT and PD was detected, whereby LCE family genes appeared to play the most important role. Within the limitations of the data analysis, autoimmunity and bone metabolisms seem to be the most relevant pathways linking the two diseases.

## Figures and Tables

**Table 1:** Datasets for HT and PD.

		PD		HT	
Datasets	GSE10334	GSE16134	GSE173078	GSE18152	GSE176153
Platform	GPL570	GPL570	GPL20301	GPL5114	GPL17303
Experimental	Array	Array	High throughput sequencing	Array	High throughput sequencing
Case sample	183	241	12	74	4
Control sample	64	69	12	6	4
Total sample	247	310	24	80	8

**Table 2:** The DEG counts of HT and PD.

	PD			HT	
Datasets	GSE10334	GSE16134	GSE173078	GSE18152	GSE176153
Analysis method	Limma	Limma	DEseq2	Limma	DEseq2
*P*value	*P* < 0.05			*P* < 0.05	
|Log2(FC)|	|Log2(FC)| >1			|Log2(FC)| >0	|Log2(FC)| >1
DEG up	152	188	90	1430	112
DEG down	42	48	473	1412	282
Total DEG	194	236	563	2842	394

**Table 3:** Topological characteristics of top 10 genes in PPI network.

Gene	MCC	Degree	DMNC	MNC	EPC	BottleNeck
LCE1B	121	6	0.64826	5	5.832	3
LCE2B	121	6	0.64826	5	5.812	3
LCE2A	120	5	0.64826	5	5.723	1
LCE2C	120	5	0.64826	5	5.751	1
LCE1C	120	5	0.64826	5	5.722	1
LCE1F	120	5	0.64826	5	5.681	1
ITGAM	25	11	0.29157	8	11.86	24
C1QB	16	7	0.38896	5	11.228	1
TREM2	12	4	0.47366	4	10.196	1
CD19	11	7	0.23775	6	10.773	4

## Data Availability

The datasets used and/or analyzed during the current study are available from the corresponding author on reasonable request.
